# High-throughput molecular identification of *Staphylococcus *spp. isolated from a clean room facility in an environmental monitoring program

**DOI:** 10.1186/1756-0500-3-278

**Published:** 2010-11-04

**Authors:** Norhan S Sheraba, Aymen S Yassin, Magdy A Amin

**Affiliations:** 1 VACSERA, The Holding Company for Biological Products & Vaccines, Giza, 22311, Egypt; 2 Department of Microbiology and Immunology, Faculty of Pharmacy, Cairo University, Cairo, 11562, Egypt

## Abstract

**Background:**

The staphylococci are one of the most common environmental isolates found in clean room facility. Consequently, isolation followed by comprehensive and accurate identification is an essential step in any environmental monitoring program.

**Findings:**

We have used the API Staph identification kit (bioMérieux, France) which depends on the expression of metabolic activities and or morphological features to identify the *Staphylococcus *isolates. The API staphylococci showed low sensitivity in the identification of some species, so we performed molecular methods based on PCR based fingerprinting of glyceraldehyde-3-phosphate dehydrogenase encoding gene as useful taxonomic tool for examining *Staphylococcus *isolates.

**Conclusions:**

Our results showed that PCR protocol used in this study which depends on genotypic features was relatively accurate, rapid, sensitive and superior in the identification of at least 7 species of *Staphylococcus *than API Staph which depends on phenotypic features.

## Background

In aseptic processing, one of the most important laboratory controls is the environmental monitoring program. This program is a defined, documented program that describes the routine particulate and microbiological monitoring of processing and manufacturing areas and includes a corrective action plan when action levels are exceeded. Environmental monitoring should promptly identify potential routes of contamination, allowing for implementation of corrections before product contamination occurs through evaluating the quality of air and surfaces in the clean room environment. The monitoring program should cover all production shifts and should include air, floors, walls, and equipment surfaces, including the critical surfaces that come in contact with the product, container, and closures. Samples should be taken throughout the classified areas of the aseptic processing facility (e.g., aseptic corridors, gowning rooms) using scientifically sound sampling procedures. Sample sizes should be sufficient to optimize detection of environmental contaminants at levels that might be expected in a given clean area.

It is important that locations posing the most microbiological risk to the product be a key part of the program. It is especially important to monitor the microbiological quality of the critical area to determine whether or not aseptic conditions are maintained during filling and closing activities. Air and surface samples should be taken at the locations where significant activity or product exposure occurs during production. Critical surfaces that come in contact with the sterile product should remain sterile throughout an operation. Critical surface sampling should be performed at the conclusion of the aseptic processing operation to avoid direct contact with sterile surfaces during processing. Detection of microbial contamination on a critical site would not necessarily result in batch rejection.

Environmental monitoring of critical and controlled areas must include a comprehensive viable monitoring program which considers the following: frequency of sampling, time at which the samples are taken (i.e., during or at the conclusion of operations), duration of sampling, sample size (e.g., surface area, air volume), specific sampling equipment and techniques, alert and action levels and appropriate response to deviations from alert or action levels.

The staphylococci are one of the most common bacterial isolates found in clean room facilities. Staphylococci are the causative agents of many opportunistic human and animal infections and are considered among the most important pathogens isolated in the clinical microbiology laboratory [1]. Coagulase-negative staphylococci (CNS) represent the majority of the species and are considered to be saprophytic or potentially pathogenic. Several species of the CNS have been involved in nosocomial infections related to implanted medical devices such as intravenous catheters, prosthetic heart valves and orthopedic implants. The species that most frequently cause diseases are *Staphylococcus epidermidis*, *Staphylococcus haemolyticus *and *Staphylococcus saprophyticus*. Other significant opportunistic pathogens include *Staphylococcus hominis*, *Staphylococcus warneri*, *Staphylococcus capitis*, *Staphylococcus simulans*, *Staphylococcus cohnii*, *Staphylococcus xylosus*, *Staphylococcus saccharolyticus*, and *Staphylococcus lugdunensis *[2,3].

Consequently, isolation followed by comprehensive and accurate identification of the distinct *Staphylococcus *species is of extreme importance in order to initiate the proper antibiotic therapy. Several methods for the identification of *Staphylococcus *spp. have been proposed. These methods usually detect traditional phenotypic properties and are available in miniaturized form for automation and convenience. The identification methods now in use range from the fully automated identification and susceptibility test systems such as the VITEK 2 (bioMérieux, France) and the BD Phoenix system (Beckton Dickinson, MD, USA) to the API Staph identification kit (bioMérieux, France) [4-6]. In addition to the previous methods, gas-liquid chromatography analysis of cellular fatty acids is also used [7]. However, methods based on phenotypic characterization are hampered by the fact that they depend on the expression of metabolic activities and or morphological features and consequently, many isolates are still poorly identified and supplementary methods are often required for complete and accurate identification.

Molecular methods such as PCR-based fingerprinting have been also used successfully for *Staphylococcus *identification at the species level [8-12]. PCR amplification of the 16 S rRNA gene, *sodA *and the glyceraldehyde-3-phosphate dehydrogenase *gap *gene has been reported [13-19]. It has been described that *Staphylococcus aureus *as well as number of coagulase-negative staphylococci, including *Staphylococcus epidermidis*, *Staphylococcus capitis*, *Staphylococcus haemolyticus *and *Staphylococcus hominis *have a 42-kDa transferrin-binding protein (Tpn) in common, located within cell wall. This protein is a member of the newly emerging family of multifunctional cell wall-associated glyceraldahyde-3-phosphate dehydrogenases which catalyze the conversion of glyceraldehyde-3-phosphate to 1, 3 diphosphoglycerate and incorporate binding sites for both transferrin and the serine protease plasmin [20-23]. Although the gene product in both *Staphylococcus saprophyticus *and *Staphylococcus warneri*, is unable to bind to human transferrin [22], the *gap* gene sequence can still be amplified by PCR. Analysis of the *gap *gene represents a high-throughput reproducible method that allows identification of distinct *Staphylococcus *species [23].

In this paper we describe a high-throughput and rapid identification method using both API Staph identification system followed by PCR analysis of the *gap *gene, for various *Staphylococcus *species isolated from environmental samples taken from air, surface, and personnel (filling, filtration and sterility areas) as a part of routine environmental monitoring program in pharmaceutical clean room facility at VACSERA (Holding Company for Biological Products & Vaccines) laboratories in Giza, Egypt.

## Materials and methods

### Sampling methods for routine environmental monitoring

#### Active air sampling

By slit-to-agar (Biological air sampler, model STA-204, New Brunswick scientific, NJ, USA) where air was drawn through slit, which rotates across the surface of an agar plate around central axis. The speed of rotation was set so that the whole surface of the plate was covered within one hour [24-26].

#### Passive air sampling

Settling plates (Petri dishes containing nutrient growth medium exposed to the environment for one hour) were used [27,28].

#### Surface and personnel sampling

Standard contact plates (RODAC: Replicate Organism Detection and Counting) were used. The convex agar meniscus allowed direct application to test surfaces (e.g. walls, floors, equipment) for hygiene control and personnel locations(hands, chest and mask). The medium used contained neutralizing agents, which inactivated any residual disinfectants on the surface to be tested and therefore enabled comparative results before and after cleaning [29-32].

#### Sampling conditions

Sampling was carried out in the operational state with process equipment running and personnel performing normal operations and in a specified condition. Sampling did not interfere with critical work zone protection or compromise the quality of any products prepared that was administered to patients. The operational condition for unidirectional airflow cabinets/isolators and transfer devices was considered to be where an operator was working in any part of the clean air device. Sampling at the rest of the facility condition was continued at an agreed frequency to monitor baseline contamination levels. The operational conditions and the activities being performed at the time of testing were recorded.

#### Sampling locations

Samples were taken from air, surface and personnel filling, filtration and sterility laboratory areas. Care was taken that samples were taken from critical and adjacent areas.

#### Culture media used

Environmental monitoring samples were grown on Tryptone Soy Agar medium, (TSA), (Bacto, France). The media was modified and contained neutralizing agents (sodium thiosulphate and lecithin) to inactivate residual surface disinfectant present on the surface to be tested.

#### Incubation conditions

Plates were incubated at 30°C - 35°C (inverted) for at least 2 days to detect bacteria and at 20°C - 25°C for at least 5 days to detect mould and fungi.

#### Interpretation of the test results

After appropriate incubation, microbiological contamination was detected by observing colonies that were enumerated as colony forming units (cfu) on each plate. Separate colony counts were tabulated for mould and bacteria.

Note: For plates used in the slit to agar sampler, cfu reading was corrected to cfu/m^3 ^according to standardized equations.

#### Bacterial strains and growth conditions

Bacterial strains used in this study were isolated from air, surface, and personnel in a pharmaceutical clean room and were kept at -80°C in glycerol stock form.

Reference strains used PCR amplification of *gap *gene were *Staphylococcus aureus *ATCC 6538 used as positive control and *Pseudomonas aergenosa *ATCC 9027 used as a negative control.

### Identification of environmental isolates by commercial Identification system API Staph

Preparation of the Strip was done following the standard procedure (bioMérieux, France). Briefly, strains were sub-cultured on Columbia Blood Agar (or P Agar), (Oxoid, England), 18-24 hours at 37°C, and checked for purity. All isolates were subjected to Gram stain to check for morphology and to confirm that they belong to the Micrococcaceae family. A homogenous suspension of each bacterial isolate was prepared in the supplied API Staph medium with turbidity corresponding to 0.5 McFarland standard. The suspension was used immediately.

The micro-tubes of the API strip were filled with the bacterial suspension; mineral oil was added to the micro-tubes that needed to be incubated under anaerobic conditions. The lid was placed and the incubation box (lid and tray) was incubated at 37°C for 20-24 hours. After the incubation period the color was observed in each micro-tube and compared to the negative control. Appropriate reagents were added as required to certain reaction and as described by the kit manual. Identification was carried using the API Staph identification software (bioMérieux, France).

### Chromosomal DNA isolation

One loopful from each glycerol stock of each isolate was streaked on TSA (Tryptic Soy Agar), (Bacto, France), plate to obtain well isolated colonies. Plates were incubated at 35°C - 37°C for 18-24 h. After incubation, one colony was inoculated into 3 ml LB broth and incubated overnight at 37°C. On the next day, the cultures were centrifuged at 4,000 g for 10 minutes at room temperature. Genomic DNA was extracted by using the EaZy Nucleic Acid Isolation Bacterial DNA Kit (Omega Bio-tech) following the instructions manual. The final DNA was eluted with water and the concentration of the DNA was determined at 260 nm using the spectrophotometer (Biotech Engineering, 80 DV UV/VIS, UK).

### PCR analysis

PCR amplification reactions were performed using a pair of primers selected on the basis of the *gap *gene nucleotide sequence of *Staphylococcus aureus *(933-bp long, from the Genbank database under accession number AJ133520). A 26-nucleotide forward primer: GF-1 (5'-ATGGTTTTGGTAGAATTGGTCGTTTA-3'), corresponding to positions 22 to 47 of the *gap *gene, and a 25-nucleotide reverse primer, GR-2 (5'- GACATTTCGTTATCATACCAAGCTG-3'), corresponding to positions 956 to 932 were selected. Primers were synthesized by (Alpha DNA, Montreal, Quebec, Canada). PCR amplification was carried out using DNA thermal cycler (Eppendorf, Gradient, Hamburg, Germany) by using PCR kit (PuReTaq Ready-To-Go™ PCR Beads from GE Healthcare, USA) following the manufacturer instructions. Briefly, the reaction contained 100 pmoles of each primer (in 0.1 μl volumes), 0.75 to 1 μg of template DNA (in 1 μl volumes), added to one tube of stable beads and water to 25 μl. The stable beads contain stabilizers, BSA (Bovine Serum Albumin), (dATP, dCTP, dGTP, and dTTP), ~ 2.5 units of pure Taq DNA polymerase and reaction buffer. DNA was denatured at 94°C for 10 minutes. This was followed by 40 cycles of DNA denaturation at 94°C for 30 sec, primer annealing at 55°C for 30 sec and extension at 72°C for 1 min. After the final cycle, reactions were terminated by an extra cycle at 72°C for 5 minutes. To visualize the product, 8 μl of each PCR reaction were mixed with 3 μl 6× loading dye (GenBioscience) and loaded on 1% Agarose gel: (Metaphor Agarose) was used for fine separation and resolution of small nucleic acids. A 100 bp DNA ladder (GenBioscience) was used as a marker.

### Statement of Ethical Approval

All experiments, involving any samples taken from personnel and done in this study, were done in accordance and approval of the ethical committee at Cairo University, Cairo, Egypt.

In addition, all personnel who contributed any samples, (swabs or any other form), did this according to their informed consent.

## Results and Discussion

Identification of environmental isolates from air, surface, personnel by using API Staph system:

A total of 43 isolates were tested using the API Staph test system (biomerieux, France). 23 isolates were taken from surfaces of filling areas (filling machine panels), sterility area (Laminar Air Flow Buttons).15 isolates were taken from active air filling areas (filtration, filling, capping, sterility and Fedagarie room (autoclave room used for sterilization of gowns and different items used in the production process). Five samples were taken from personnel. The isolates were taken according to a routine environmental monitoring program.

Most of the isolates drawn from surface represent 65% of total isolates; the samples comply with the European commission guide due to GMP.

The result of the API Staph identification showed that among the 23 isolate taken from surfaces almost half of them (12 isolates) were identified as *Staphylococcus hominis*, three isolates were identified as *Staphylococcus epidermidis*, two isolates of each of *Staphylococcus xylosus *and *Staphylococcus aureus*, and one isolate of each of *Staphylococcus warneri*, *Staphylococcus sciuri*, *Staphylococcus lugdunensis*, and *Staphylococcus haemolyticus*.

Among the 15 samples taken from air, more than half (8 isolates) were identified as *Staphylococcus hominis*, four isolates were identified as *Staphylococcus haemolyticus*, and one of each of *Staphylococcus epidermidis*, *Staphylococcus lugdunensis *and *Staphylococcus warneri*.

Isolates taken from personnel were identified as two being *Staphylococcus hominis*, two isolates as *Staphylococcus epidermidis *and one isolate as *Staphylococcus aureus*

The result of the API Staph identification is summarized in table 1 which shows the different species of *Staphylococcus *isolated from each main source (surface, personnel or air) and the % identity of each isolate.

**Table 1:** Different species of *Staphylococcus *and their % identity

API Identification	Number of isolates	% identity
*Staphylococcus hominis*	22	47.3%-80%

*Staphylococcus epidermidis*	6	89%-97.8%

*Staphylococcus haemolyticus*	5	41%-89.9%

*Staphylococcus aureus*	3	69%-99%

*Staphylococcus lugdunensis*	2	42%-64.7%

*Staphylococcus warneri*	2	38%-74.7%

*Staphylococcus xylosus*	2	90%-99.8%

*Staphylococcus sciuri*	1	27.3%

Total	43	

The result shows that almost half of the total isolates (22 isolates) regardless of origin were identified as *Staphylococcus hominis*, a coagulase-negative member of the bacterial genus *Staphylococcus*.

*Staphylococcus hominis *occurs very commonly as a harmless commensal on human and animal skin, which explains its abundance among the isolates from different sources. However, like many other coagulase-negative staphylococci, *S. hominis *may occasionally cause infection in patients whose immune system is compromised, for example by chemotherapy or predisposing illness.

A total of six isolates were identified as *Staphylococcus epidermidis *which is also coagulase-negative and occurs frequently on the skin of humans and animals and in mucous membranes. Due to contamination, *S. epidermidis *is probably the most common species found in laboratory tests which explains its abundance among the isolates. *S. epidermidis *is usually non-pathogenic, it is an important cause of infection in patients whose immune system is compromised, or who have indwelling catheters. Many strains produce a biofilm that allows them to adhere to the surfaces of medical prostheses.

A total of five isolates were identified as *Staphylococcus haemolyticus *a coagulase-negative, and catalase positive staphylococci, frequently found as a commensal organism on the skin of humans and animals, *S. haemolyticus *occurs infrequently as a cause of soft-tissue infections, usually in immunocompromised patients.

*S. haemolyticus *is resistant to multiple antimicrobial agents. Resistance to vancomycin has been recorded, and this is a cause for concern because such resistance could be acquired by other, more pathogenic staphylococci [33].

Three isolates were identified as *Staphylococcus aureus*, the most common cause of Staph infections. It is frequently living on the skin or in the nose of a person. Approximately 20-30% of the general populations are "Staph carriers".

*Staphylococcus aureus *can cause a range of illnesses from minor skin infections, such as pimples, impetigo, boils, cellulites, furuncles, carbuncles, scalded skin syndrome and abscesses, to life-threatening diseases, such as pneumonia, meningitis, osteomyelitis, endocarditis, toxic shock syndrome (TSS), and septicemia [34].

Other Staphylococcus species identified in this study as *S. xylosus*, *S. warneri*, *S. lugdunensis *and *S. sciuri *are all common commensals on human skin and mucous membranes. Consequently, the identity of the *Staphylococcus *isolates showed a typical distribution in an environmental sample with the majority of the isolates being those commonly found as commensals on skin surfaces.

**Figure 1:**
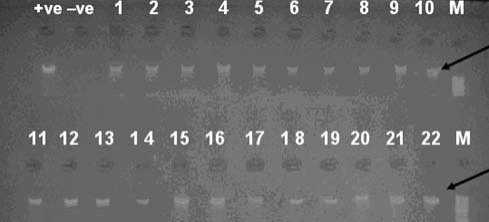
**PCR products of isolates numbered 1 to 22**. Agarose gel electrophoresis of 933-bp PCR amplification products from chromosomal DNA from staphylococcal species using primers GF-1 and GR-2, M: Ladder marker, black arrow refers to the correct size product of the gap gene corresponding to 933 base pairs. +ve: *Staphylococcus aureus *ATCC 6538,-ve: *Pseudomonas aeruginosa *ATCC 9027. Samples 1-22 except 3&4: *Staphylococcus hominis*, 3: *Staphylococcus aureus *and 4: *Staphylococcus sciuri*.

**Figure 2:**
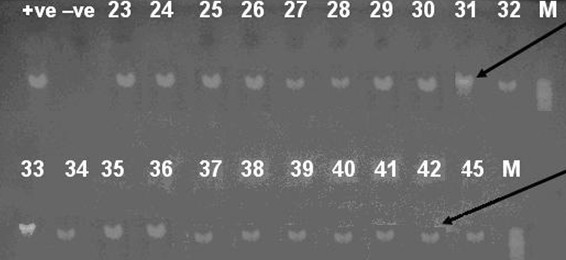
**PCR products of isolates numbered 23 to 45**. Agarose gel electrophoresis of 933-bp PCR amplification products from chromosomal DNA from staphylococcal species using primers GF-1 and GR-2, M, +ve, -ve and black arrow are the same as in figure 1. Samples 23, 45: *Staphylococcus hominis*, 24-29: *Staphylococcus epidermidis*, 30, 31: *Staphylococcus aureus*, 32, 33: *Staphylococcus lugdunensis*, 34, 35: *Staphylococcus warneri*, 36, 37: *Staphylococcus xlyosus and *38-42: *Staphylococcus haemolyticus*.

35 out of the 43 isolates (81%) identified using API Staph identification system showed identity range between (57.1%-99.8%). Although the majority of the isolates were in a "good identification" range (70%), several isolates showed a relatively weak or poor identification. We decided to investigate the clones on a molecular level by attempting to amplify the glyceraldehyde-3-phosphate dehydrogenase *gap *gene. The *gap *gene has proved to be a very well conserved gene that can be used as a useful tool in PCR assays for identification of *Staphylococcus *species [15]. The *gap *gene product glyceraldehyde-3-phosphate dehydrogenase has been discovered to be located within the cell walls of *S. aureus *and other coagulase-negative staphylococci. The pair of primers used in this study successfully primed the synthesis of a 933 bp fragment corresponding to the *gap* gene. All the isolates gave the corresponding PCR band equivalent to the *gap* gene product as seen after agarose gel electrophoresis (Figures 1 and 2) confirming the identity of the isolates as belonging to the genus *Staphylococcus*. All the *Staphylococcus *species identified in our study (table 1) were similarly identified with PCR amplification of the *gap *gene in previous studies [15,19,35] confirming the reproducibility, reliability and efficiency of the method.

In conclusion, we used the API Staph identification system followed by PCR amplification of the *gap *gene to identify various *Staphylococcus *species isolated from a clean room environment. The methods showed a reliable and rapid identification and can be used for the analysis of large number of samples in a high-throughput manner.

## Consent

Written informed consent was obtained from the personnel that donated samples for publication of this manuscript and accompanying images. A copy of the written consent is available for review by the Editor-in Chief of this journal.

## Competing interests

The authors declare that they have no competing interests.

## Authors' contributions

NSS carried out the experimental procedure. ASY supervised and participated in the experimental procedure and drafted the manuscript. MAA designed the study. All authors read and approved the final manuscript.

## References

[B1] Fidalgo S Vazquez F Mendoza MC Perez F Mendez FJ Bacteremia due to Staphylococcus epidermidis: microbiologic, epidemiologic, clinical, and prognostic features Rev Infect Dis 1990 12 3 520 528 2359910 10.1093/clinids/12.3.520

[B2] Calvo J Hernandez JL Farinas MC Garcia-Palomo D Aguero J Osteomyelitis caused by Staphylococcus schleiferi and evidence of misidentification of this Staphylococcus species by an automated bacterial identification system J Clin Microbiol 2000 38 10 3887 3889 11015429 10.1128/jcm.38.10.3887-3889.2000 PMC87502

[B3] Shuttleworth R Behme RJ McNabb A Colby WD Human isolates of Staphylococcus caprae: association with bone and joint infections J Clin Microbiol 1997 35 10 2537 2541 9316903 10.1128/jcm.35.10.2537-2541.1997 PMC230006

[B4] Ieven M Verhoeven J Pattyn SR Goossens H Rapid and economical method for species identification of clinically significant coagulase-negative staphylococci J Clin Microbiol 1995 33 5 1060 1063 7615705 10.1128/jcm.33.5.1060-1063.1995 PMC228104

[B5] Miller JM Biddle JW Quenzer VK McLaughlin JC Evaluation of Biolog for identification of members of the family Micrococcaceae J Clin Microbiol 1993 31 12 3170 3173 8308109 10.1128/jcm.31.12.3170-3173.1993 PMC266370

[B6] Watts JL Washburn PJ Evaluation of the Staph-Zym system with staphylococci isolated from bovine intramammary infections J Clin Microbiol 1991 29 1 59 61 1993769 10.1128/jcm.29.1.59-61.1991 PMC269703

[B7] Stoakes L John MA Lannigan R Schieven BC Ramos M Harley D Hussain Z Gas-liquid chromatography of cellular fatty acids for identification of staphylococci J Clin Microbiol 1994 32 8 1908 1910 7989541 10.1128/jcm.32.8.1908-1910.1994 PMC263901

[B8] Cuny C Witte W Typing of Staphylococcus aureus by PCR for DNA sequences flanked by transposon Tn916 target region and ribosomal binding site J Clin Microbiol 1996 34 6 1502 1505 8735106 10.1128/jcm.34.6.1502-1505.1996 PMC229050

[B9] Goh SH Santucci Z Kloos WE Faltyn M George CG Driedger D Hemmingsen SM Identification of Staphylococcus species and subspecies by the chaperonin 60 gene identification method and reverse checkerboard hybridization J Clin Microbiol 1997 35 12 3116 3121 9399505 10.1128/jcm.35.12.3116-3121.1997 PMC230133

[B10] Kumari DN Keer V Hawkey PM Parnell P Joseph N Richardson JF Cookson B Comparison and application of ribosome spacer DNA amplicon polymorphisms and pulsed-field gel electrophoresis for differentiation of methicillin-resistant Staphylococcus aureus strains J Clin Microbiol 1997 35 4 881 885 9157147 10.1128/jcm.35.4.881-885.1997 PMC229695

[B11] Mendoza M Meugnier H Bes M Etienne J Freney J Identification of Staphylococcus species by 16S-23 S rDNA intergenic spacer PCR analysis Int J Syst Bacteriol 1998 48 Pt 3 1049 1055 10.1099/00207713-48-3-1049 9734063

[B12] Marcos JY Soriano AC Salazar MS Moral CH Ramos SS Smeltzer MS Carrasco GN Rapid identification and typing of Staphylococcus aureus by PCR-restriction fragment length polymorphism analysis of the aroA gene J Clin Microbiol 1999 37 3 570 574 9986814 10.1128/jcm.37.3.570-574.1999 PMC84472

[B13] Becker K Harmsen D Mellmann A Meier C Schumann P Peters G von Eiff C Development and evaluation of a quality-controlled ribosomal sequence database for 16 S ribosomal DNA-based identification of Staphylococcus species J Clin Microbiol 2004 42 11 4988 4995 10.1128/JCM.42.11.4988-4995.2004 15528685 PMC525259

[B14] Fontana C Favaro M Pelliccioni M Pistoia ES Favalli C Use of the MicroSeq 500 16 S rRNA gene-based sequencing for identification of bacterial isolates that commercial automated systems failed to identify correctly J Clin Microbiol 2005 43 2 615 619 10.1128/JCM.43.2.615-619.2005 15695654 PMC548051

[B15] Yugueros J Temprano A Berzal B Sanchez M Hernanz C Luengo JM Naharro G Glyceraldehyde-3-phosphate dehydrogenase-encoding gene as a useful taxonomic tool for Staphylococcus spp J Clin Microbiol 2000 38 12 4351 4355 11101563 10.1128/jcm.38.12.4351-4355.2000 PMC87604

[B16] Poyart C Quesne G Boumaila C Trieu-Cuot P Rapid and accurate species-level identification of coagulase-negative staphylococci by using the sodA gene as a target J Clin Microbiol 2001 39 12 4296 4301 10.1128/JCM.39.12.4296-4301.2001 11724835 PMC88539

[B17] Sivadon V Rottman M Chaverot S Quincampoix JC Avettand V de Mazancourt P Bernard L Trieu-Cuot P Feron JM Lortat-Jacob A Use of genotypic identification by sodA sequencing in a prospective study to examine the distribution of coagulase-negative Staphylococcus species among strains recovered during septic orthopedic surgery and evaluate their significance J Clin Microbiol 2005 43 6 2952 2954 10.1128/JCM.43.6.2952-2954.2005 15956429 PMC1151921

[B18] Blaiotta G Casaburi A Villani F Identification and differentiation of Staphylococcus carnosus and Staphylococcus simulans by species-specific PCR assays of sodA genes Syst Appl Microbiol 2005 28 6 519 526 10.1016/j.syapm.2005.03.007 16106559

[B19] Ghebremedhin B Layer F Konig W Konig B Genetic classification and distinguishing of Staphylococcus species based on different partial gap, 16 S rRNA, hsp60, rpoB, sodA, and tuf gene sequences J Clin Microbiol 2008 46 3 1019 1025 10.1128/JCM.02058-07 18174295 PMC2268370

[B20] Modun BJ Cockayne A Finch R Williams P The Staphylococcus aureus and Staphylococcus epidermidis transferrin-binding proteins are expressed in vivo during infection Microbiology 1998 144 Pt 4 1005 1012 10.1099/00221287-144-4-1005 9579074

[B21] Modun B Evans RW Joannou CL Williams P Receptor-mediated recognition and uptake of iron from human transferrin by Staphylococcus aureus and Staphylococcus epidermidis Infect Immun 1998 66 8 3591 3596 9673237 10.1128/iai.66.8.3591-3596.1998 PMC108390

[B22] Modun B Kendall D Williams P Staphylococci express a receptor for human transferrin: identification of a 42-kilodalton cell wall transferrin-binding protein Infect Immun 1994 62 9 3850 3858 8063401 10.1128/iai.62.9.3850-3858.1994 PMC303040

[B23] Modun B Williams P The staphylococcal transferrin-binding protein is a cell wall glyceraldehyde-3-phosphate dehydrogenase Infect Immun 1999 67 3 1086 1092 10024547 10.1128/iai.67.3.1086-1092.1999 PMC96433

[B24] Gosden PE MacGowan AP Bannister GC Importance of air quality and related factors in the prevention of infection in orthopaedic implant surgery J Hosp Infect 1998 39 3 173 180 10.1016/S0195-6701(98)90255-9 9699136

[B25] Leeming JP Pryce-Roberts DM Kendrick AH Smith EC The efficacy of filters used in respiratory function apparatus J Hosp Infect 1995 31 3 205 210 10.1016/0195-6701(95)90067-5 8586789

[B26] Ho J Spence M Duncan S An approach towards characterizing a reference sampler for culturable biological particle measurement Journal of Aerosol Science 2005 36 5-6 557 573 10.1016/j.jaerosci.2004.11.020

[B27] Pollok NL Williams GH Shay DE Barr CE Laminar air purge of microorganisms in dental aerosols J Am Dent Assoc 1970 81 5 1131 1139 4918661 10.14219/jada.archive.1970.0384

[B28] Kelkar U Kelkar S Bal AM Kulkarni S Kulkarni S Microbiological evaluation of various parameters in ophthalmic operating rooms. The need to establish guidelines Indian J Ophthalmol 2003 51 2 171 176 12831148

[B29] Lemmen SW Häfner H Zolldann D Amedick G Lutticken R Comparison of two sampling methods for the detection of Gram-positive and Gram-negative bacteria in the environment: moistened swabs versus Rodac plates Int J Hyg Environ Health 2001 203 3 245 248 10.1078/S1438-4639(04)70035-8 11279821

[B30] Faure O Fricker-Hidalgo H Lebeau B Mallaret MR Ambroise-Thomas P Grillot R Eight-year surveillance of environmental fungal contamination in hospital operating rooms and haematological units J Hosp Infect 2002 50 2 155 160 10.1053/jhin.2001.1148 11846544

[B31] Favero MS Puleo JR Marshall JH Oxborrow GS Comparative levels and types of microbial contamination detected in industrial clean rooms Appl Microbiol 1966 14 4 539 551 5954479 10.1128/am.14.4.539-551.1966 PMC546777

[B32] Poletti L Pasquarella C Pitzurra M Savino A Comparative efficiency of nitrocellulose membranes versus RODAC plates in microbial sampling on surfaces J Hosp Infect 1999 41 3 195 201 10.1016/S0195-6701(99)90016-6 10204121

[B33] Froggatt JW Johnston JL Galetto DW Archer GL Antimicrobial resistance in nosocomial isolates of Staphylococcus haemolyticus Antimicrob Agents Chemother 1989 33 4 460 466 2729941 10.1128/aac.33.4.460 PMC172460

[B34] Chambers HF The changing epidemiology of Staphylococcus aureus? Emerg Infect Dis 2001 7 2 178 182 10.3201/eid0702.010204 11294701 PMC2631711

[B35] Layer F Ghebremedhin B Konig W Konig B Differentiation of Staphylococcus spp. by terminal-restriction fragment length polymorphism analysis of glyceraldehyde-3-phosphate dehydrogenase-encoding gene J Microbiol Methods 2007 70 3 542 549 10.1016/j.mimet.2007.06.015 17681623

